# Supplementary material for: Associations between humiliation, shame, self-harm and suicidal behaviours among adolescents and young adults: A systematic review protocol
Source: PLoS One. 2022 Nov 23;17(11):e0278122. doi: 10.1371/journal.pone.0278122 (PMC9683542; doi:10.1371/journal.pone.0278122)
Supplement: S1 File — (DOCX) [file pone.0278122.s002.docx]

**Appendix 1**

**Search strategies in PubMed**

| Search terms |
| --- |
| Search: (humiliation) AND "Self-Injurious Behavior"[Mesh] Filters: Adolescent: 13-18 years, Young Adult: 19-24 years Sort by: Most Recent  (("humiliate"[All Fields] OR "humiliated"[All Fields] OR "humiliating"[All Fields] OR "humiliation"[All Fields] OR "humiliations"[All Fields]) AND "Self-Injurious Behavior"[MeSH Terms]) AND (adolescent[Filter] OR youngadult[Filter]) |
| Search: (humiliation) AND ( "Suicide"[Mesh] OR "Suicide, Attempted"[Mesh] OR "Suicide, Completed"[Mesh] ) Filters: Adolescent: 13-18 years, Child: 6-12 years, Young Adult: 19-24 years Sort by: Most Recent (("humiliate"[All Fields] OR "humiliated"[All Fields] OR "humiliating"[All Fields] OR "humiliation"[All Fields] OR "humiliations"[All Fields]) AND ("Suicide"[MeSH Terms] OR "suicide, attempted"[MeSH Terms] OR "suicide, completed"[MeSH Terms])) AND (adolescent[Filter] OR child[Filter] OR youngadult[Filter]) |
| Search: **("Shame"[Mesh]) OR "Embarrassment"[Mesh]) OR "Harassment, Non-Sexual"[Mesh]) NOT ( "Bullying"[Mesh] OR "Cyberbullying"[Mesh] )) AND "Self-Injurious Behavior"[Mesh])** Filters: **Adolescent: 13-18 years, Young Adult: 19-24 years**  ((("Shame"[MeSH Terms] OR "Embarrassment"[MeSH Terms] OR "harassment, non sexual"[MeSH Terms]) NOT ("Bullying"[MeSH Terms] OR "Cyberbullying"[MeSH Terms])) AND "Self-Injurious Behavior"[MeSH Terms]) AND (adolescent[Filter] OR youngadult[Filter]) |
| Search: **shame [Mesh] OR embarrassment [Mesh] OR ''harassment non-sexual'' [Mesh]NOT bullying [Mesh] AND suicide [Mesh]** Filters: **Adolescent: 13-18 years, Young Adult: 19-24 years** Sort by: **Most Recent**  ((("shame"[MeSH Terms] OR "embarrassment"[MeSH Terms] OR "harassment, non sexual"[MeSH Terms]) NOT "bullying"[MeSH Terms]) AND "suicide"[MeSH Terms]) AND (adolescent[Filter] OR youngadult[Filter]) |
| Search: **shame [Mesh] OR embarrassment [Mesh] OR ''harassment non-sexual'' [Mesh]NOT bullying [Mesh] AND suicide [Mesh]** Filters: **Adolescent: 13-18 years, Young Adult: 19-24 years** Sort by: **Most Recent**  ((("shame"[MeSH Terms] OR "embarrassment"[MeSH Terms] OR "harassment, non sexual"[MeSH Terms]) NOT "bullying"[MeSH Terms]) AND "suicide"[MeSH Terms]) AND (adolescent[Filter] OR youngadult[Filter]) |
| Search: **shame [Mesh] OR embarrassment [Mesh] OR ''harassment non-sexual'' [Mesh]NOT bullying [Mesh] AND self-harm*** Filters: **Adolescent: 13-18 years, Young Adult: 19-24 years** Sort by: **Most Recent**  ((("shame"[MeSH Terms] OR "embarrassment"[MeSH Terms] OR "harassment, non sexual"[MeSH Terms]) NOT "bullying"[MeSH Terms]) AND "self harm*"[All Fields]) AND (adolescent[Filter] OR youngadult[Filter]) |
| Search: **shame [Mesh] OR embarrassment [Mesh] OR ''harassment non-sexual'' [Mesh]NOT bullying [Mesh] AND ‘’ intentional self-injury’’** Filters: **Adolescent: 13-18 years, Young Adult: 19-24 years** Sort by: **Most Recent**  ((("shame"[MeSH Terms] OR "embarrassment"[MeSH Terms] OR "harassment, non sexual"[MeSH Terms]) NOT "bullying"[MeSH Terms]) AND ("self injurious behavior"[MeSH Terms] OR ("self injurious"[All Fields] AND "behavior"[All Fields]) OR "self injurious behavior"[All Fields] OR ("intentional"[All Fields] AND "self"[All Fields] AND "injury"[All Fields]) OR "intentional self injury"[All Fields])) AND (adolescent[Filter] OR youngadult[Filter]) |
| Search: shame [Mesh] OR embarrassment [Mesh] OR ''harassment non-sexual'' [Mesh]NOT bullying [Mesh] AND overdose Filters: Adolescent: 13-18 years, Young Adult: 19-24 years  ((("shame"[MeSH Terms] OR "embarrassment"[MeSH Terms] OR "harassment, non sexual"[MeSH Terms]) NOT "bullying"[MeSH Terms]) AND ("drug overdose"[MeSH Terms] OR ("drug"[All Fields] AND "overdose"[All Fields]) OR "drug overdose"[All Fields] OR "overdose"[All Fields] OR "overdoses"[All Fields] OR "overdosed"[All Fields] OR "overdosing"[All Fields])) AND (adolescent[Filter] OR youngadult[Filter]) |
| Search: shame [Mesh] OR embarrassment [Mesh] OR ''harassment non-sexual'' [Mesh]NOT bullying [Mesh] AND ‘’ deliberate self-poisoning’’ Filters: Adolescent: 13-18 years, Young Adult: 19-24 years  ((("shame"[MeSH Terms] OR "embarrassment"[MeSH Terms] OR "harassment, non sexual"[MeSH Terms]) NOT "bullying"[MeSH Terms]) AND (("deliberate"[All Fields] OR "deliberated"[All Fields] OR "deliberately"[All Fields] OR "deliberates"[All Fields] OR "deliberating"[All Fields] OR "deliberation"[All Fields] OR "deliberations"[All Fields]) AND "self poisoning"[All Fields])) AND (adolescent[Filter] OR youngadult[Filter]) |
| Search: **shame [Mesh] OR embarrassment [Mesh] OR ''harassment non-sexual'' [Mesh]NOT bullying [Mesh] AND ‘’ non-suicidal self-injury’’** Filters: **Adolescent: 13-18 years, Young Adult: 19-24 years**  ((("shame"[MeSH Terms] OR "embarrassment"[MeSH Terms] OR "harassment, non sexual"[MeSH Terms]) NOT "bullying"[MeSH Terms]) AND ("self injurious behavior"[MeSH Terms] OR ("self injurious"[All Fields] AND "behavior"[All Fields]) OR "self injurious behavior"[All Fields] OR ("non"[All Fields] AND "suicidal"[All Fields] AND "self"[All Fields] AND "injury"[All Fields]) OR "non suicidal self injury"[All Fields])) AND (adolescent[Filter] OR youngadult[Filter]) |
| Search: **shame [Mesh] OR embarrassment [Mesh] OR ''harassment non-sexual'' [Mesh]NOT bullying [Mesh] AND ‘’ self-mutilation’’** Filters: **Adolescent: 13-18 years, Young Adult: 19-24 years**  ((("shame"[MeSH Terms] OR "embarrassment"[MeSH Terms] OR "harassment, non sexual"[MeSH Terms]) NOT "bullying"[MeSH Terms]) AND ("self mutilation"[MeSH Terms] OR ("self"[All Fields] AND "mutilation"[All Fields]) OR "self mutilation"[All Fields])) AND (adolescent[Filter] OR youngadult[Filter]) |
| Search: **shame [Mesh] OR embarrassment [Mesh] OR ''harassment non-sexual'' [Mesh]NOT bullying [Mesh] AND ‘’ suicidal thought’’** Filters: **Adolescent: 13-18 years, Young Adult: 19-24 years**  ((("shame"[MeSH Terms] OR "embarrassment"[MeSH Terms] OR "harassment, non sexual"[MeSH Terms]) NOT "bullying"[MeSH Terms]) AND (("suicid"[All Fields] OR "suicidal"[All Fields] OR "suicidality"[All Fields] OR "suicidally"[All Fields] OR "suicidals"[All Fields] OR "suicide"[MeSH Terms] OR "suicide"[All Fields] OR "suicides"[All Fields] OR "suicide s"[All Fields] OR "suicided"[All Fields] OR "suiciders"[All Fields]) AND ("thinking"[MeSH Terms] OR "thinking"[All Fields] OR "thought"[All Fields] OR "thoughts"[All Fields] OR "thought s"[All Fields] OR "thoughtful"[All Fields] OR "thoughtfulness"[All Fields]))) AND (adolescent[Filter] OR youngadult[Filter]) |
| Search: **shame [Mesh] OR embarrassment [Mesh] OR ''harassment non-sexual'' [Mesh]NOT bullying [Mesh] AND ‘’ intentional self-injury’’** Filters: **Adolescent: 13-18 years, Young Adult: 19-24 years** Sort by: **Most Recent**  ((("shame"[MeSH Terms] OR "embarrassment"[MeSH Terms] OR "harassment, non sexual"[MeSH Terms]) NOT "bullying"[MeSH Terms]) AND ("self injurious behavior"[MeSH Terms] OR ("self injurious"[All Fields] AND "behavior"[All Fields]) OR "self injurious behavior"[All Fields] OR ("intentional"[All Fields] AND "self"[All Fields] AND "injury"[All Fields]) OR "intentional self injury"[All Fields])) AND (adolescent[Filter] OR youngadult[Filter]) |
| Search: **shame [Mesh] OR embarrassment [Mesh] OR ''harassment non-sexual'' [Mesh]NOT bullying [Mesh] AND ‘’ suicidal intent’’** Filters: **Adolescent: 13-18 years, Young Adult: 19-24 years**  ((("shame"[MeSH Terms] OR "embarrassment"[MeSH Terms] OR "harassment, non sexual"[MeSH Terms]) NOT "bullying"[MeSH Terms]) AND (("suicid"[All Fields] OR "suicidal"[All Fields] OR "suicidality"[All Fields] OR "suicidally"[All Fields] OR "suicidals"[All Fields] OR "suicide"[MeSH Terms] OR "suicide"[All Fields] OR "suicides"[All Fields] OR "suicide s"[All Fields] OR "suicided"[All Fields] OR "suiciders"[All Fields]) AND ("intention"[MeSH Terms] OR "intention"[All Fields] OR "intent"[All Fields] OR "intentions"[All Fields] OR "intentional"[All Fields] OR "intentioned"[All Fields] OR "intents"[All Fields]))) AND (adolescent[Filter] OR youngadult[Filter]) |
| Search: **shame [Mesh] OR embarrassment [Mesh] OR ''harassment non-sexual'' [Mesh]NOT bullying [Mesh] AND ‘’ suicidal ideation’’** Filters: **Adolescent: 13-18 years, Young Adult: 19-24 years**  ((("shame"[MeSH Terms] OR "embarrassment"[MeSH Terms] OR "harassment, non sexual"[MeSH Terms]) NOT "bullying"[MeSH Terms]) AND ("suicidal ideation"[MeSH Terms] OR ("suicidal"[All Fields] AND "ideation"[All Fields]) OR "suicidal ideation"[All Fields])) AND (adolescent[Filter] OR youngadult[Filter]) |
| Search: self-harm OR intentional self-injury OR deliberate self-harm OR overdose OR deliberate self-poisoning OR non-suicidal self-injury OR self-mutilation OR suicidal thought OR suicidal ideation OR suicidal intent AND shame Filters: Child: 6-12 years, Adolescent: 13-18 years, Young Adult: 19-24 years |

**Search strategies in other data bases (**Web of Science Core Collection, CINAHL, PsycINFO, Embase, and ProQuest)

| ( Self-injurious behaviour OR suicide OR suicided attempted OR suicide completed OR self-harm OR intentional self-injury OR deliberate self-harm OR overdose OR deliberate self-poisoning OR non-suicidal self-injury OR self-mutilation OR suicidal thought OR suicidal ideation OR suicidal intent OR suicide ) AND abasement AND ( children or adolescents or youth or child or teenager ) |
| --- |
| ( Self-injurious behaviour OR suicide OR suicided attempted OR suicide completed OR self-harm OR intentional self-injury OR deliberate self-harm OR overdose OR deliberate self-poisoning OR non-suicidal self-injury OR self-mutilation OR suicidal thought OR suicidal ideation OR suicidal intent OR suicide ) AND victimisation AND ( children or adolescents or youth or child or teenager ) |
| ( Self-injurious behaviour OR suicide OR suicided attempted OR suicide completed OR self-harm OR intentional self-injury OR deliberate self-harm OR overdose OR deliberate self-poisoning OR non-suicidal self-injury OR self-mutilation OR suicidal thought OR suicidal ideation OR suicidal intent OR suicide ) AND degradation AND ( children or adolescents or youth or child or teenager ) |
| ( Self-injurious behaviour OR suicide OR suicided attempted OR suicide completed OR self-harm OR intentional self-injury OR deliberate self-harm OR overdose OR deliberate self-poisoning OR non-suicidal self-injury OR self-mutilation OR suicidal thought OR suicidal ideation OR suicidal intent OR suicide ) AND harassment AND ( children or adolescents or youth or child or teenager ) |
| ( Self-injurious behaviour OR suicide OR suicided attempted OR suicide completed OR self-harm OR intentional self-injury OR deliberate self-harm OR overdose OR deliberate self-poisoning OR non-suicidal self-injury OR self-mutilation OR suicidal thought OR suicidal ideation OR suicidal intent OR suicide ) AND embarrassment AND ( children or adolescents or youth or child or teenager ) |
| ( Self-injurious behaviour OR suicide OR suicided attempted OR suicide completed OR self-harm OR intentional self-injury OR deliberate self-harm OR overdose OR deliberate self-poisoning OR non-suicidal self-injury OR self-mutilation OR suicidal thought OR suicidal ideation OR suicidal intent OR suicide ) AND shame AND ( children or adolescents or youth or child or teenager ) |
| ( Self-injurious behaviour OR suicide OR suicided attempted OR suicide completed OR self-harm OR intentional self-injury OR deliberate self-harm OR overdose OR deliberate self-poisoning OR non-suicidal self-injury OR self-mutilation OR suicidal thought OR suicidal ideation OR suicidal intent OR suicide ) AND humiliation AND ( children or adolescents or youth or child or teenager ) |
