# Supplementary material for: Associations between humiliation, shame, self-harm and suicidal behaviours among adolescents and young adults: A systematic review protocol
Source: PLoS One. 2022 Nov 23;17(11):e0278122. doi: 10.1371/journal.pone.0278122 (PMC9683542; doi:10.1371/journal.pone.0278122)
Supplement: Attachment — Submitted filename: Response to the reviewers AMcL.docx [file pone.0278122.s003.docx]

| Reviewer (s) suggestion | Our response | Changes have made in the manuscript | Section/paragraph |
| --- | --- | --- | --- |
| Is it not the obj 2 should come first? | We agree with the suggestion | Objective 2 moved into the place of objective 1 | Objectives |
| Please consider providing a table containing the search command using Boolean operators. | Details of the search strategy has now been included as a supplementary file. | -- | Supplementary file |
| - Since authors mention to run moderation analysis, please kindly consider stating what potential moderators authors are expecting to be a potential moderator/s. It may be my lack of understanding that I am not clear about what authors mean by ".......considering population characteristics and methodological aspects of studies". How does a methodological approach is relevant to the moderation analysis? Plus what sub-group analysis author are expecting to run? and what would be the population sub-type? A little description may increase my understanding. | We revised the text and provided more clarity on moderation analysis and subgroup analysis. | Moderation analyses may be conducted to understand the potential association with the effect sizes. For instance, to analyse the potential moderating effects of sex on humiliation or shame, the effect size of studies that consisted of females will be compared with the effect sizes of the studies that consisted of males.  If possible, sub-group analyses will be conducted according to the population subtypes *(clinical vs non-clinical and/or children vs young adults.)* | Data Synthesis |
| Please kindly consider describing the process of GRADE | We have included more details on the process of GRADE. | If there is sufficient evidence to make recommendations, we will rate the certainty of evidence based on Cochrane methods and in accordance with the Grading of Recommendations Assessment, Development and Evaluation (GRADE)[52]. The GRADEpro will be used to construct a Summary of Findings. Accordingly, the certainty of evidence will be graded as high, moderate, low, and very low based on the five domains including risk of bias, imprecision, inconsistency, indirectness, and publication bias. | Data Synthesis |
| Do the authors plan for present the meta-analysis by adolescent and young adults since they are two distinct age groups? Plus their experience/ understanding of Humiliation, Shame, way of Self-Harm etc. might be much different. I don't see any rationale of using such two distinct age groups in the introduction except to see that ".....adolescents and young adults has not been well examined". | We have included one paragraph to discuss the rationale for including the two distinct age groups.  There is an absence of studies published in relation to humiliation and shame and the suicidal process in children. As such, it has been necessary to extend our review to incorporate young adults. We acknowledge the difference in the humiliation/shame experience across each distinct group and reference this in our review.  In addition, this subgroup variation will be taken into account while analysing and interpreting the results. | The rationale for selecting two distinct age groups (adolescents’ and young adults’) for this review is based on the increased prevalence of self-harm repetition among these age groups in recent years, with some interesting age and gender variations [48]. Whilst the self-harm repetition rate was high among adolescent females (15-19 year old), in comparison to young adults; this repetition rate was also high among young adult males [48]. | Introduction/background |
| Reviewer #1  I appreciate the effort of the review authors. I found the protocol very well written. The introduction and discussion sections are excellent. Please find my comments in the methods section. Methods: Eligibility criteria (Study design): Qualitative studies including case reports or case series will be excluded.” – Please rephrase as “Qualitative studies, case reports, and case series will be excluded.” | Thank you. We have rephrased the sentence as per reviewer suggestion. | Qualitative studies, case reports, and case series will be excluded. | Methods-Eligibility criteria |
| Reviewer 1  Information sources: “The literature search will be conducted from 20th September 2021 to 29th April 2022.” – This is the search timing I guess. Please mention the search timeline/ period (for example: from inception till April 2022, or from 2000 to April 2022”) | We have included the details of the search timeline. | The studies will be searched from inception to 29^th^ April 2022. | Methods- information sources |
| Reviewer-1  Search strategy: “Based on our initial scoping review via electronic databases, the following MESH terms or key/index terms were identified.” – What do you mean by scoping review? As a scoping review is a specific type of systematic review, it’s creating confusion. Did the author mean “initial exploration”? | We have rephrased the sentence. | Based on our initial search of the electronic databases, the following MESH terms or key/index terms were identified. | Methods – Search Strategy |
| Reviewer-1  Search strategy: As per the PRISMA 2020 guidelines, a comprehensive search strategy for all databases should be provided (at least as a supplementary file). Please provide a comprehensive search strategy for all databases. | Yes, we have included the search strategy as a supplementary file | --- | Supplementary file |
| Reviewer 1  Full-text screening: Please mention that the full-text screening will be conducted by two independent review authors | Yes, we have mentioned these details now. | Two of the review authors (AS & AMcL) will then screen the full text reports and decide whether these meet the inclusion criteria. | Methods-Study selection process |
| Reviewer 1  Will the data be extracted by two independent review authors? If yes, please mention it. If not, please explain | Yes. We have mentioned these details now. | Data will be extracted by two independent review authors (AMcL & AS). | Methods-Data collection process |
| Risk of bias assessment: “The studies will be assessed for methodological quality using the Joanna Briggs Institute (JBI) critical appraisal tools for analytical cross-sectional (eight-item) [50] and cohort studies (eleven-item) [51]” – Previously authors have mentioned that they will include cross-sectional studies, case-control studies, cohort studies, experimental/ quasi-experimental studies (if they mention baseline data). Now, why only two JBI checklists have been mentioned? JBI has got separate checklists for separate study designs. Mention all as applicable. | We have included the details of JBI-Case control appraisal tool.  Since the baseline data only be collected from the experimental or quasi experimental studies, we do not anticipate using a separate risk assessment tool for this category of studies. We will use the JBI - cross sectional critical appraisal. | The studies will be assessed for methodological quality using the Joanna Briggs Institute (JBI) critical appraisal tools for analytical cross sectional (eight-item) [51], cohort studies (eleven-item) [52] or Case Control Studies (ten-item)[53] | Methods- Risk of bias assessment section |
| Reviewer #2: This is a well described protocol with a solid rationale, and I believe the work will make a valuable contribution to knowledge. I look forward to seeing the results of the review when it is complete. | Thank you. |  |  |
